# Professionalism experiences of undergraduate learner nurses during their 4-year training programme at a Higher Education Institution in the Western Cape, South Africa

**DOI:** 10.4102/curationis.v42i1.2030

**Published:** 2019-10-28

**Authors:** Portia Bimray, Karien Jooste, Hester Julie

**Affiliations:** 1 School of Nursing, University of the Western Cape, Cape Town, South Africa; 2 Department of Nursing, Cape Peninsula University of Technology, Cape Town, South Africa; 3 Faculty of Community Health Science, University of the Western Cape, Cape Town, South Africa

**Keywords:** Higher Education Institution, nursing education, undergraduate student nurses, professionalism, professional values, nurse training programme

## Abstract

**Background:**

Professional socialisation of student nurses needs to be integrated into the formal teaching and learning during the nursing programme. Embedded in the training programme are professional values that are used synonymously with nursing professionalism. Professionalism is the conduct, qualities, values, vision, mission and/or goals that characterise a profession, and describes behaviours that are expected within the profession’s members. However, one’s values are shaped by one’s experiences, influence one’s behaviour and interactions with others, and are manifested in many aspects of professional behaviour. New nurses to the profession are expected to display behaviours of professionalism, thus requiring nurse training schools to help students internalise these behaviours. Nurse educators therefore carry a responsibility to shape future nurses’ growth towards professionalism.

**Objectives:**

This article reports on the experiences of undergraduate student nurses regarding nursing professionalism during their 4-year training programme at a Higher Education Institution in the Western Cape, South Africa.

**Method:**

A qualitative, exploratory and descriptive design was applied. Eight focus group discussions were conducted with first- to fourth-year student nurses registered for the undergraduate nursing programme. Data were transcribed verbatim and analysed using open coding. Ethical principles and trustworthiness were maintained throughout the study.

**Results:**

Six main themes indicated that undergraduate student nurses experienced issues with role modelling, language barriers, their own understanding of professional behaviour, reasons for students and practitioners’ unprofessional behaviour, prejudice towards degree students and students’ professional or unprofessional behaviour experienced as contributing to the image of the profession.

**Conclusion:**

Student nurses received mixed messages leading to emotional turbulence. They needed clear guidance from role models to demonstrate how to behave professionally.

## Introduction

Nursing professionalism is an integral part of the formal curriculum of the undergraduate nursing programme leading to a professional qualification. Embedded in the training programme are professional values, a term which is used synonymously with nursing professionalism (Jooste 2017:21). Professional nursing values refer to the attitudes, beliefs and priorities of nurses that ultimately function as a guide and motivation in interactions with patients, colleagues and other professionals (Leners, Roehrs & Piccone 2006 in Caldwell & Miller 2016). In the context of the nursing profession, values are essential to maintain high standards of nursing care (Kaya et al. 2017:716). It is generally assumed that student nurses who have completed an accredited training programme and have been socialised into the professional culture would have internalised the requisite professional values (Jooste 2017:22). These values are important to healthcare facilities because they are an important way of living out ethical commitment and affect patient safety and outcomes (Caldwell & Miller 2016:3). Caldwell and Miller (2016:3) mention that because prospective employers and practising nurses are looking for professional values in new nurses, nursing schools and nursing students should be concerned with student development of professional nursing values.

## Background

A review of literature at international and national levels demonstrates the challenges of nursing students in basing their clinical practices on professional values. Research has focused on seeking patterns to explain deficiencies in values that lead to a diminished quality of patient care (Caldwell & Miller 2016). The increase in ethical conflicts in healthcare necessitates further addressing of professional values (Shafakhah et al. 2018:5).

Developing professional values in nursing students strengthens their capacity for reasoning and ethical decision-making in challenging situations and enables the provision of safe, legal and ethical care. It is also an important aspect of nursing education, which is taught through training nursing students and clinical experiences (Shafakhah et al. 2018:5). According to Shafakhah, these values develop through education and contact with nurse educators. The challenges to the development of professional values are a key concern for nursing educators.

Nursing educators are mindful of the challenges students are faced with in practice. Cognisance is taken of factors such as the burden of disease, shortages of nurses, cultural diversity and a stressed socio-economic environment, which could impact professional nursing practice and health service delivery (Erasmus 2008:62) (Muller 2009:75). The current healthcare environment requires competent professional nurses capable of managing complex professional issues. It thus becomes essential for professional nurses to take responsibility for their actions, as well as initiative to understand and master the changing work situations, yet to keep up with the increasing demands and changes in healthcare practice (De Braganca & Nirmala 2017:60).

Coupled with the above factors is the overall professional culture of a student’s training environment, which is a powerful determinant in shaping professional values and attitudes, and which is believed to contradict or work against what is learned in the classroom. Training programmes must aim to provide professionals with professional values that will enable them to make a meaningful contribution to health services and the practice environment to improve patient care outcomes (South African Government Gazette 2011). Geyer, Mogotlane and Young (2010:33) are of the opinion that the student nurse entering the profession should positively commit to adjusting his or her behaviour in the interests of ensuring quality and safe patient care. This may require adaptation of the personal values of student nurses to ensure that their conduct adheres to the professional values of nursing. Caldwell and Miller (2016:37) indicate that it is possible for student nurses to transition from a lay to a professional nurse over the course of their 4 years. It is, hence, undoubtedly that awareness of the need for strong professional values on the part of nurse educators, clinical facilitators and professional nurses is needed in preparing nurses to manage patient care in a capable and professional manner. By developing these professional values, a student nurse develops her or his professional identity (Fagermoen 1997:434).

The development of professional values and behaviours forms a significant part of the professional socialisation process (Hammer 2000:455). Professional socialisation is a process through which people selectively acquire the current values and attitudes, interests, skills and knowledge (culture) in the groups in which they are, or groups of the profession of which they seek to become a member (Hammer 2000:455).

Kubsch, Hansen and Huyser-Eatwell (2008:375) state that despite the gains that have been achieved regarding the professionalisation of nursing, the profession continues to struggle with its professional status. Kubsch et al. (2008) agree that nursing education programmes at institutions of higher education play a significant role in how professional values are learned and demonstrated in nursing practice. It is argued that the educational preparation of the registered nurse (RN) may make a difference in professional values, as nursing education programmes instil in their student nurses the notion that they are professionals and members of the nursing profession (Kubsch et al. 2008:375). The authors suggest that these values acquired in nursing education programmes may be discarded after the nurse has graduated. Some of the reasons for this may be that values were imposed on the student rather than freely chosen, or because of work pressures. Iacobucci et al. (2012:418) confirm that the internalisation of professional nursing values among undergraduate nurses continuously increases from junior to senior levels during their training. Despite findings which indicate that professional value internalisation evolves during undergraduate nursing programmes, student nurses continue to report difficulty in enacting behaviours that support values because of various professional and organisational factors (Iacobucci et al. 2012).

## Theoretical framework

This study was guided by the theoretical framework of Brown and Ferrill’s (2009:4) taxonomy of professionalism. Brown and Ferrill (2009) created a professionalism model to organise the abstract concept of professionalism and reflect how professional values link with professional behaviours. Generally, value development is a long-term process of consolidating and embedding one’s own beliefs, attitudes and values into one’s moral behaviour. Any kind of human development is associated with learning, for example, learning ideas and skills, and then making use of them. These factors were considered in the data analysis and interpretation of the findings. The model of Brown and Ferrill (2009:4) assumes a focus shift from learning to practical performance or behaviour. Assumptions about professionalism in nursing that emanate from the model were adapted for this study. The elements of professionalism include the core professional values (also referred to as characteristics) of nursing. Professional values of nurses are, thus, linked to their expected professional behaviour in practice. A student’s professional values determine his or her professional behaviour in nursing practice. The development of professional values is a long-term process of learning that leads to professional behaviour in a professional practice setting.

## Research methods and design

### Aim

This study aims to report on the experiences of undergraduate student nurses regarding professionalism during their 4-year training programme at a Higher Education Institution (HEI) in the Western Cape. The study was part of a bigger research project that developed a conceptual framework for nurse educationalists and professional nurses to facilitate professionalism among undergraduate student nurses at a HEI in the Western Cape.

### Study design

This was an exploratory, descriptive and contextual qualitative study design. Eight focus group discussions (FGDs) were used to explore data from student nurses regarding nursing professionalism in an undergraduate programme in the Western Cape.

### Setting

The study was conducted at a HEI that offers a 4-year degree programme for nurses in the Western Cape. The programme consists of an integrated theory–practice component. Theoretical learning takes place in a classroom environment, while student nurses are placed for clinical learning at accredited public healthcare facilities across the Western Cape Metropole.

### Study population and sampling strategy

The target population was all undergraduate student nurses (1058) registered for the B.NUR programme in 2013 in a school of nursing at a HEI in the Western Cape. Purposive sampling was followed with first- to fourth-year student nurses. Any participants who did not give consent to partake in the study, or who did not meet the inclusion criteria, were excluded from the study.

### Data collection

Student FGDs were used as a means of data collection. The FGDs were held during the students’ lunch hour, or after classes. The FGD sessions lasted for not longer than 60 min. During this time, participants were encouraged to ask questions about the study. An interview guide was used to pose the questions. The initial question asked to student nurses was: ‘What are your experiences about professionalism in the undergraduate nursing programme at this institution?’.

### Data analysis

Recorded data were transcribed verbatim. All the transcripts were carefully read and reviewed to contribute to a meaningful whole. The data were subsequently analysed using a process of identifying themes in the data for underlying meanings. Categories were assigned to the themes, and the researcher also reviewed documented observations made during the interviews. A total of eight focus group interviews were conducted until data saturation occurred and no new data emerged. No new information was observed at the eighth focus group of student nurses.

### Ethical considerations

Ethical approval to conduct this study was obtained from Research Ethics Committee of a university in the Western Cape and permission was granted by the Western Cape Department of Health to conduct the study with participants at the accredited healthcare facilities where the learner nurses were placed for clinical learning. Ethical clearance registration number 12/10/18. Ethical principles and trustworthiness were maintained throughout the study. The following ethical principles were adhered to

- the principles of no harm and beneficence
- the principle of respect and justice
- the principle of privacy and confidentiality.

## Results

Despite being exposed to and trained to develop professionalism and receiving support from educationalists and professional nurses (both in theory and practice), the student nurses experienced mixed messages and emotional turbulence about what it really means to be a professional. The dominant themes that emerged from the data were role modelling of unprofessional behaviour; language barriers in the development of professional behaviour; participants’ own understanding of professional behaviour; reasons for students and practitioners’ unprofessional behaviour and suggestions for improvement; prejudice towards degree students; and students’ professional or unprofessional behaviour experienced as contributing to the image of the profession.

### Role modelling of unprofessional behaviour

The findings reflected strong feelings about the ‘unprofessional behaviour’ of professional nurses in some settings and wards towards the university’s first- and second-year students. Examples of poor role modelling or misconduct were mentioned, while some participants advocated for students to take control of negative role modelling and pay attention to their own unprofessional behaviour. The experiences of unprofessional behaviour by nurse practitioners included humiliating and demoralising communication towards student nurses.

The dominant experience related to professional nurses’ communication with students. These experiences were regarded as not upholding the values of respect and dignity of co-workers or peers: ‘she’s shouting in front of the patients, your dignity is going to like decrease’ (1st year, P11, FG1).

A participant described being humiliated in front of peer student nurses by the way the nurse educationalist communicated loudly with her:

‘I also had a misunderstanding with a supervisor and the way she conducted herself with me. She was not; … I can say and stand for this, that she was not professional. She insulted me in front of my peers and she spoke loud enough so that her colleagues could also hear.’ (1st year, P7, FG2)

Participants expressed the need for professional nurses to demonstrate to them how to behave professionally in the clinical placements:

‘I believe that the sisters in the wards have a huge role to play to portray how professionalism should be and … practically showing us how to behave professional. We do have lots of information – theoretically … you can teach me how to remove sutures, but it is a different story to remove them. You must show me how to remove them also if you’re going to teach me. I need someone to show me how to behave professionally and that is not happening.’ (4th year, P3, FG3)

### Language barriers in the development of professional behaviour

Student nurses throughout all year levels in the undergraduate nursing programme experienced that language barriers negatively affected their professional development. They had difficulty in understanding the language in the placement facilities because of the way people spoke with each other:

‘I think there is a language barrier. … It must come with the way you talk to people. … There is a language barrier there. We don’t understand.’ (4th year, P6, FG3)

On the contrary, occurrences were described where participants were treated with kindness, and where professional staff were considerate in showing that they cared about the cultural differences of student nurses, by explaining tasks to them in a language that they understood:

‘… The environment that I’m working in, the nurses there are very professional, even though they are Afrikaans speaking. But they’ve got a way of making you feel comfortable, even though their files/folders are written in Afrikaans. But they are willing to explain and try to make you understand what does this mean.’ (1st year, female P, FG1)

Participants also described that not being able to express themselves in a specific language lowered their self-image:

‘And English. Because when you’re not able to communicate, you become shy when you need to address anyone – whether it’s a superior or someone else, a co-student, you become inferior to address them because you feel that, okay; I’m not fluent in this kind of speech.’ (1st year, female P, FG2)

### Participants’ understanding of professional behaviour

Participants thought that professional behaviour should be centred around admiration for educators and mutual respect between educators and students:

‘Mutual respect. One needs to make sure mutual respect. I think that’s the first … I think that’s the most important thing. Just as long as there’s respect for the teacher and respect for the students as well.’ (3rd year, P1, FG2)

Participants mentioned that background played a role by indicating that nurses enter the nursing profession with basic values of respect, kindness, politeness or good manners, and punctuality, and that they should maintain these values throughout the undergraduate nursing programme:

‘… In your schooling and in your life experience you just learn the basic values, punctuality – when you have to be somewhere, you must be there, you must be on duty. You know if you have an appointment, you must be there. Basic respect, basic politeness, all of these things are just basic human conduct that most people have. And it just seems that for some reason when you become a student you just lose all of these values.’ (4th year, P3, FG3)

### Reasons for students and practitioners’ unprofessional behaviour and suggestions for improvement

It seems that a lack of comprehension of professionalism resulted in undesirable behaviour on the part of the participants. Student nurses revealed that they memorised the professional values to pass an assessment:

‘I didn’t even understand it. I just memorised it and then I wrote it in a test and then I let it go.’ (4th year, P6, FG3)

Participants were unclear about what professionalism meant and how they should apply it in the clinical settings:

‘Not everyone knows what the word “professionalism” means. They don’t know. So they act, like in their eyes, they’re professional. But actually they’re not. Because there’s not enough emphasis placed on professionalism. Also, in the clinical settings you get to work in, 20 percent of the time the staff is professional, but the other 80 percent of the time they’re not. They do what they want to do and that is the time that you also fall into that trap when you do things that isn’t right. And that is unprofessional.’ (4th year, P4, FG3)

There was a need for clear guidance and direction on how to act professionally in nursing practice, and the value of having standards of professionalism:

‘And I would really appreciate it if there should be someone who can explain to me what must I do. If you want professionalism, you must put everything down. What must I do if you want me to be a professional – 100 percent professional.’ (4th year, P6, FG3)

They were unaware whether professional standards exist:

‘ … If there should be standards of professionalism for us to be taught. What are the standards? What are the things if I’m doing this I’m being unprofessional? Or if I’m doing this I’m not being professional. I think that will help us a lot to understand what professionalism is all about. Honestly, we are told to be professional, but we don’t know how to be professional.’ (4th year, P2, FG3)

Other participants thought that a mentor should be available to whom they could air their personal issues:

‘I think if we can just have someone like a mentor to go to just to in regard to any questions that you would have, just to make sure that you are still fine and on track, not just your studies, but your personal life as well. It would be much easier … at least once a month.’ (3rd year, P4, FG2)

### Practitioners’ prejudice towards degree students

There was a perception among the hospital staff that university degree student nurses thought that they were better at performing skills:

‘So they always have that mind-set that the university students think that they’re smarter than them because we do things in a certain way.’ (3rd year, P1, FG1)

However, other participants felt judged and discriminated against because of the HEI from which they came. They expressed feelings of inferiority and humiliation in clinical settings:

‘You’re from HEI A. You don’t know what you do … and … they would be like why are you so stupid? They are very condescending and judgmental towards you.’ (3rd year, P1, FG2)

Participants also mentioned having issues with the professional nurse in charge, who passed judgement and compared the practical skills of the university students to those of the college students in the clinical practice setting:

‘The sister in charge … they have like that attitude, HEI A (offering degree) students are full of theory and not practical. So they’re like … they are separating us. No, the HEI B (offering non-degree) students are like good in practical and you are the ones that are good in theory. So we don’t want theory here at work.’ (1st year, P3, FG1)

### Students’ professional or unprofessional behaviour was experienced as contributing to the image of the profession

According to participants, some student nurses did not know how to conduct themselves professionally and were disrespectful towards senior professional staff in the clinical placement settings:

‘They (some students) don’t know how to speak to the other person, how to speak to a senior when they’re working as well. So they don’t know how to be professional or what to do to act professional towards any other person.’ (4th year, P1, FG3)

It was reported that student nurses were not serious and did not have the appropriate attitude or mind-set when they went to the nursing workplace environment:

‘… We are taking this student mentality to a hospital or to a work place and we’re still thinking we’re students in that sense – have fun and enjoy ourselves throughout the whole day, which is not true. Immediately there is a professional standard in the hospital self.’ (3rd year, P1, FG2)

## Discussion

This qualitative study reports on the experiences of undergraduate student nurses regarding nursing professionalism during their training at a HEI. The experiences of student nurses in an undergraduate programme highlight important issues for both nursing education and nursing practice. Undergraduate student nurses, throughout their 4-year training programme, have expressed some of the main aspects that could be factors in preventing them from reaching the expected nursing professionalism at the end of their training.

Role modelling has been highlighted as essential in undergraduate training programmes for student nurses. Role models who portray a positive attitude and are approachable play a vital role in supporting these student nurses in the classroom and clinical learning environment. Important learning, including the teaching of concepts, theory, critical thinking skills and research, happens in the classroom, but is best integrated with the skills learned in the clinical setting where integration of theory and practice takes place (Cunze & Van Rensburg 2016). Role models as leaders are needed to guide followers appropriately, so that they feel inspired and motivated to carry out the right tasks (Zhu et al. 2012). It is regarded as essential that professional leaders set a positive example as role models whose attitudes and values are assimilated by students. The most appropriate way for students to learn what it means to be professional is to see it in action (Felstead 2013).

Language barriers can cause limitations and challenges to healthcare professionals (Omolola & Gray 2014:18). Within the context of South Africa as a diverse country with different languages, findings in this study revealed that language was a barrier to communication in the clinical placement facilities, and that especially English could be an obstacle in the professional development of student nurses during their undergraduate training programme. English as a second language complicates communication in the clinical practice environment (Ho & Coady 2018). Student nurses from diverse cultural backgrounds often misunderstand or misinterpret messages in practice, when English is not their first or preferred language of communication. This is consistent with a finding by Houle (2010) that the majority of nurses reported that language barriers are a significant impediment to quality care and a source of stress in the workplace. Incomplete nursing assessments, misunderstood medical information and the lack of therapeutic relationships between providers and patients are problems encountered when there is limited English proficiency among role players (Butts 2013:93; Houle 2010). In this study, participants reported on the use of Afrikaans as the language of choice in healthcare facilities in the Western Cape Metropole. The patient’s treatment plan should be communicated in a language universal to all healthcare professionals involved with the patient. Furthermore, professional nurses should convey body language that is congruent with their verbal communication because they are sending out a professional message that has great impact on the emotions experienced by student nurses (Diogo et al. 2016). Student nurses become more receptive to the messages of the professional nurse if they can sense true caring in the body language of the professional nurse.

Professional behaviours influence nursing professionalisation and these behaviours strongly impact professional development (Tanaka et al. 2016). Professional behaviour was understood by student nurses as having important values such as respect and dignity to interact with others in a caring way. Being treated with respect has a positive impact on trust and the self-image of a person. Poor interpersonal relationships with clinical staff are seen as barriers to learning, and may have a huge impact on students’ attitudes and confidence (Lawal et al. 2015). Communication that humiliates students is regarded as negative and creates effects such as anxiety in the learning environment (Richmond, Wrench & Gorham 2009). Diogo et al. (2016) allude to the importance of monitoring the emotional needs of students in clinical practice.

The need for emotional support of student nurses therefore becomes essential, as it has been indicated that student nurses often experience emotional trauma in different forms during their placement in the clinical facilities (Diogo et al. 2016). A supportive learning environment is expected by these student nurses where they feel comfortable enough to make mistakes and learn from them. They learn from mentors who appreciate their individuality and different learning needs. Nurse educators must therefore ensure that the clinical environment is ideal for learning (Lawal et al. 2015). The provision of emotional support to student nurses should, thus, be by nurse educationalists in the academic environment, who connect with them on a different level in the classroom setting and who understand their unique needs, as opposed to the professional nurses in the clinical learning environment. Although the university or enrolling institution does provide support structures for students with academic and psychosocial needs, these resources are available to the broader campus community. However, student nurses have expressed that their need for support is different from the general student population on campus.

The reasons for unprofessional behaviour that were reported in the findings of this study were, among others, the lack of understanding of what professionalism means, that the specifics of professionalism are not clear and that there is a need for standards and practical guidelines. It would be invaluable if student nurses could be empowered by nurse educationalists and professional nurses to resist poor practice examples, by having clear professional policies and guidelines in place that would guide the student nurse in the clinical placement facilities on how to behave in the practice environment. Consistent with the previous work by Laschinger, Zhu and Read (2016), the student nurses need support and guidance as they transition into their professional role. This is essential for new nurses learning their professional role, and especially for those student nurses who may be unsure of their scope of practice. Professional nurses should guide student nurses to adhere and conform to the rules of the profession, to achieve in the student nurse the desired standard of what is acceptable and unacceptable behaviour in clinical practice. Adherence to guidelines (e.g. procedural performances) promotes patient safety and is associated with positive clinical outcomes (Nilsson et al. 2014:1). It is, however, not an unusual phenomenon that deviation from guideline practices does occur during a student nurse’s training. The issue of students deviating from practice guidelines while attending a university programme should be taken seriously (Nilsson et al. 2014:6). As such, it has been confirmed in this study that professional values are taught but not internalised by undergraduate student nurses. Disparities between idealism and professionalism taught in nursing education programmes in a classroom environment may be part of the challenges experienced by the new student nurses when exposed to the realities of the clinical practice environment. Often a perfect situation is sketched in a classroom setting, which is not reflective of the real-life situation in practice. Therefore, providing ongoing support in the form of mentorship for student nurses is important to ensure that they are receiving the necessary guidance in learning how to be professional (Laschinger et al. 2016).

The findings also indicated differences in perceptions of student nurses who enter the clinical learning environment from different training institutions. Participants who undergo the undergraduate training at the university, for degree purposes, are occasionally labelled as ‘being full of theory and not practical’ and compared with their counterparts who study at nursing colleges for non-degree qualifications when they are in clinical placement facilities, sharing the accredited facilities with nursing students from other institutions. Furthermore, undergraduate student nurses from the university felt judged and discriminated against by the attitudes of hospital staff. They have expressed feelings of inferiority when they are in the facilities supported by the following responses: ‘You’re from university A. You don’t know what you do’ (1st year, P1, FG2) and ‘Why are you so stupid? They are very condescending and judgmental towards you’ (3rd year, P1, FG2).

They struggle to fit in with perceived skilled student nurses, who study at the college and who are placed for a longer period of time in the practice environment during their training. The time spent in the wards or facilities by college students leads to formation of relationships or comfort in the practical setting that the undergraduate student nurses from the university do not have the luxury of developing. However, increased nursing experience is not associated with increased professional values (Caldwell & Miller 2016:5). While the perception is that college nurses perform better because they are more exposed to practical work in their training, degree nurses undergo an integrated theory–clinical curriculum that encourages critical thinking. There is, thus, an assumption among permanent hospital staff that degree nurses ‘know more’, and therefore the expectations are higher for student nurses who are in the undergraduate training programme. The findings suggest that undergraduate student nurses experience challenges in the clinical learning environment. Similar challenges have been reported in previous studies, in that clinical learning is always influenced by relationships in context, that is, student–staff relationships are shaped by the curricular and programme context (Jamshidi et al. 2016). Not being exposed to clinical learning in the same way as college-trained nursing students, for example, created a specific situation for student nurses in degree programmes. These dynamics created conditions that constrained learning. Equality and non-judgemental treatment of training nurses from different nurse training institutions at healthcare facilities are essential for achieving learning outcomes and gaining competence in theory and practice.

The study revealed that some student nurses did not act with respect and did not take professional responsibility in practice seriously. Student nurses are expected to apply a different mind-set or attitude in clinical practice by conducting themselves appropriately in the different learning environments to when they are in the classroom setting on campus. When they go as ordinary students to the workplace environment, student nurses should be aware of the professional standards that exist in the hospitals. These standards should be adhered to when they transition from the academic learning environment. However, participants reported that they wanted to be regarded as normal university students who want to have fun or feel free to be like the bigger student community who seems to be carefree students on campus. Their peers were of the opinion that this mind-set should change when they enter the clinical practice environment, as student nurses should know how to conduct themselves professionally in practice. Their communication with senior professional nurses in the clinical placement settings should be respectful. Participants also strongly motivated for student nurses to take responsibility for their own learning. Peer student nurses verbalised the importance for student nurses to have the professional capability to create a learning environment in which they take ownership of being competent in initiating their own learning, and that they are self-motivated while conveying a positive image of the nursing profession.

### Strengths and limitations

This study formed part of a larger doctoral study that describes the experiences regarding nursing professionalism from the perspectives of three cases, that is, student nurses, nurse educationalists and professional nurses. They remain the main stakeholders in nursing practice and nursing education. The views of student nurses are therefore the same under contextual conditions and beliefs about professionalism within nursing education and practice. The original contribution of the study is its relevance to nursing education and nursing practice in preparing nursing graduates for their future careers and service in a dynamic, yet unpredictable environment, in which the professional values of the nursing profession could be compromised. Furthermore, the study provides input into the implementation of the Provincial Nursing Strategy to address challenges faced by the nursing profession in the Western Cape in facilitating professionalism in professional nursing practice. New knowledge was generated by a framework that included all stakeholders involved with the education of the new generation nurse, to internalise professional values in an ever-changing environment. This article reports on the integration of the findings from the case of student nurses into the professionalism framework. The qualitative study emphasises depth and insight from the information produced by undergraduate students and cannot be generalised to the larger population of student nurses across the Western Cape.

The study is also limited to HEIs and healthcare facilities in the Western Cape Metropole, focusing on a setting in an urban area in which nursing education and clinical practice take place. Future research should focus on rural areas, other urban areas and other provinces as this would provide an understanding of the extent of the phenomena around nursing professionalism across the entire country, taking into consideration a wider contextual field to explore.

### Implications and recommendations

The issue of nursing professionalism remains a topic of wide discussion. It continues to lack a clear definition to guide new comers to the profession on how to behave when they do not have role models to demonstrate what it means to be professional.

The following recommendations are suggested as a result of the findings. Professional nurses in clinical nursing practice should *inter alia* role model the total image, the code of conduct and the expected professional behaviours required for nursing practice, and demonstrate the desired attitudes, values and behaviours of professionalism expected from all members of the profession. They should provide a proper orientation for undergraduate student nurses to ensure that they know what is expected of them from the start and that they get a sense of belonging or feeling welcome as part of the team of professional nurses in practice. The orientation programme for new nurses should include concepts in the nurses’ pledge and code of conduct or ethics. It is strongly recommended that professional nurses provide privacy when student nurses are disciplined for their mistakes in the patient care wards and foster a culture in which student nurses feel comfortable to ask questions or look for help when they are in the clinical placement environment.

It is furthermore recommended that educational opportunities be provided to accommodate the different and specific learning needs of both undergraduate student nurses and those student nurses from non-degree programmes. Student nurses from the respective programmes, however, should be treated equally at similar levels of learning and should be treated respectfully for the strengths they bring to the clinical learning environment in practice.

Healthcare institutions need to ensure that the communication policy in all hospitals includes the use of a universal language that will prevent misunderstandings that might potentially be an obstacle in achieving excellence in patient care outcomes.

Lastly, healthcare institutions should make the expectations that will facilitate professionalism among student nurses known. There is, thus, a need for wards within the healthcare institution to make the vision, mission and goals that lead to excellence in patient care visible to all nursing staff.

## Conclusions

This study reported on the experiences of undergraduate student nurses regarding professionalism during their training at a HEI in the Western Cape Metropole. Undergraduate student nurses are expected to internalise professional values and assume nursing professionalism during their training programme at a university or HEI. Challenges that they experience regarding nursing professionalism have significance for both nursing education and nursing practice.
